# The Associations between the Number of School Sports Teams That a Student Regularly Participates in and Factors Such as Perceived Stress, Loneliness, and Sleep Satisfaction among Korean Adolescents Who Have Attempted Suicide

**DOI:** 10.3390/children11010077

**Published:** 2024-01-09

**Authors:** Jeonga Kwon, Jusun Jang

**Affiliations:** 1 Department of Elementary Education, College of First, Korea National University of Education, Cheongju-si 28173, Republic of Korea; gilddong1234555@knue.ac.kr; 2 Department of Sports Science, Education Research Industry Cluster at Ansan (ERICA) Campus, Hanyang University, Ansan-si 15588, Republic of Korea

**Keywords:** adolescents, loneliness, perceived stress, sleep satisfaction, sports team, suicide

## Abstract

Adolescents who have attempted suicide are more likely to experience a recurrence of suicidal behavior, thus necessitating systematic follow-ups and management. We aimed to investigate the association between the number of school sports teams that a student regularly participates in and psychological factors such as perceived stress, loneliness, and sleep satisfaction among 1393 Korean adolescents who have attempted suicide by using data from the 2022 Korea Youth Risk Behavior Survey. Frequency analyses were performed to evaluate participant characteristics. Chi-square analyses were used to examine the differences in participant characteristics according to the number of teams. Multivariate logistic regression analysis was used to examine the associations between the number of teams and perceived stress, loneliness, and sleep satisfaction. Our results indicated that participating in a greater number of sports teams at school was more strongly associated with reduced perceived stress, reduced loneliness, and increased sleep satisfaction. Specifically, the odds ratio (OR) for feeling very much stressed was 0.613 (range: 0.387–0.969; *p* = 0.036) among the participants who belonged to one team. Among those who belonged to two teams, the OR for feeling very much stressed was 0.482 (range: 0.281–0.286; *p* = 0.008). Among those who belonged to two teams, the OR for feeling a lot of stress was 0.514 (range: 0.304–0.870; *p* = 0.013), and that for not feeling much stress was 2.663 (range: 1.103–6.426; *p* = 0.029). The OR for not feeling much stress was 4.697 for those who belonged to three teams (range: 1.531–14.408; *p* = 0.007) and 6.671 for those who belonged to four or more teams (range: 1.858–23.953; *p* = 0.004). The OR for feeling no stress at all was 11.629 (range: 2.229–60.661; *p* = 0.004) for those who belonged to three teams and 93.531 (range: 19.260–454.207; *p* < 0.001) for those who belonged to four or more teams. In terms of loneliness, the OR for rarely feeling lonely was 2.651 (range: 1.148–6.123; *p* = 0.022) among those who belonged to one team. The OR for feeling lonely all the time was 0.370 (range: 0.155–0.884; *p* = 0.025) among those who belonged to two teams. In terms of sleep satisfaction, the OR for having very sufficient sleep was 4.371 (range: 1.627–11.742; *p* = 0.003) for those who belonged to four or more teams. These results suggest that school sports are an advantageous tool for suicide prevention, given their low costs, absence of side effects, and ease of participation.

## 1. Introduction

Suicide represents a serious global public health concern, with 703,000 people dying by suicide each year worldwide, and remains the fourth leading cause of death among adolescents aged 15–19 years [1]. Since 2003, South Korea (Korea hereafter) has ranked first in suicide deaths among the 35 member countries of the Organisation for Economic Cooperation and Development, and suicide is considered the leading cause of death among Korean adolescents. As such, there is no expectation that the problem of youth suicide will be easily solved [2]. Suicidal ideation is a complex phenomenon influenced by the interaction of many factors (e.g., biological, psychological, sociocultural, and family-related factors). Depression, parental divorce, suicidal behavior among friends, posttraumatic stress disorder, eating disorders, bipolar disorder, dating violence, problems with social relationships, feelings of hopelessness, substance abuse, academic stress, and sexual frustration have all been reported to contribute to suicidal ideation in adolescents [3].

Although the many physical and psychological changes that occur during adolescence can make young people prone to risky behaviors such as suicide, physical education and physical activities at school may aid in mitigating some of these risks. Several studies have demonstrated that higher levels of physical activity and active participation in school physical education classes are significantly associated with a reduction in suicide-related behaviors among adolescents [4,5,6]. Sibold et al. [6] reported that levels of suicidal ideation and numbers of suicide attempts among bullied adolescents decreased as the number of days spent participating in physical activity increased. Physical activity improves self-esteem and provides an opportunity for social support, both of which are cited as protective factors against suicidal thoughts in adolescents [7]. Physiologically, physical activity decreases levels of the stress hormone cortisol and increases the levels of neurotransmitters such as brain-derived neurotrophic factor, serotonin, dopamine, and norepinephrine; these changes may help to reduce suicidal ideation/behavior via improvements in mental health (i.e., reduced depression and increased self-esteem) [8,9]. Behaviorally, participating in school sports may prompt adolescents who are at risk of suicide to reconsider their choices because of increased self-confidence and an improved ability to overcome difficult situations [10].

School sports go beyond physical activities that are performed alone because some sports require collaboration and communication with one’s peers in a team. For adolescents, participating in team sports can strengthen communication skills and aid in maintaining friendships, and the beneficial effects of such participation on mental health appear to be greater than those observed for individual sports [11,12,13,14]. A study reported that students who participated in team sports were more likely to obtain support from friends and coaches, thus regulating their mental health and decreasing the risk of suicide attempts during difficult times [15]. This finding suggests that exercising with social support may play a vital role in suicide prevention efforts [15].

Adolescents who have attempted suicide are more likely to do so again, thus making systematic follow-ups and management essential for preventing recurrence. Although this strategy mainly involves medication and psychological counseling, medication carries a high risk of side effects such as dizziness and lethargy, and psychological counseling is less accessible depending on the place of residence [16,17]. Physical activity via participation in team sports at school represents a realistic, low-cost way to address the risk of suicidal behaviors among adolescents without the side effects associated with medication use. Perceived stress, loneliness, and sleep are known to contribute to suicidal behavior [4,5,18]. To identify potential strategies for preventing the recurrence of suicidal behavior and provide basic data that can be used for policy development, we aimed to explore the associations between the number of teams participating in school sports activities and perceived stress, loneliness, and sleep satisfaction among adolescents who have attempted suicide.

## 2. Materials and Methods

### 2.1. Study Design and Participants

The data used in this study were obtained from the 2022 Korea Youth Risk Behavior Survey (KYRBS), which was organized by the Korea Disease Control and Prevention Agency (KDCA). This annual survey collects data on the physical activity, mental health, smoking, alcohol consumption, and eating habits of adolescents, and the results are used to support policy efforts. The data used in this study were collected in October 2022, obtained voluntarily with the consent of the adolescents and their guardians, and approved by the KDCA (approval number: 117058). The adolescents used computers or mobile devices at school to participate in the survey, and the results were posted on the KDCA website (https://www.kdca.go.kr/yhs/home.jsp (accessed on 30 November 2023). The data on the website were attached to ID numbers, and all personal identifying information was removed. A total of 51,850 adolescents participated in the survey. The question, “Have you attempted suicide in the last 12 months?” was used; 50,457 participants answered “no”, while 1393 answered “yes”. Therefore, the data from 50,457 adolescents who had never attempted suicide were excluded, while those from 1393 adolescents who had attempted suicide were considered suitable and used in this study. This study was conducted according to the principles outlined in the Declaration of Helsinki.

### 2.2. Measures

The number of school sports teams in which each adolescent remained active during the semester was considered the independent variable. Accordingly, adolescents with suicidal experiences were asked to indicate the number of sports teams they belonged to, using a five-point Likert scale: 1 = no team, 2 = one team, 3 = two teams, 4 = three teams, and 5 = four or more teams. The raw data were used without any modification in this study.

Perceived stress, loneliness, and sleep satisfaction were used as the dependent variables. These were also measured using Likert scales, where scores 1 to 5 were used to measure an individual’s attitude, opinions, and thoughts about each question. First, the responses to the question, “How much stress do you usually feel?” were used to determine the level of perceived stress, using a five-point Likert scale: 1 = I feel extremely stressed; 2 = I feel a lot of stress; 3 = I feel a little stressed; 4 = I do not feel much stress; and 5 = I do not feel stressed at all). Second, the level of loneliness was determined through the responses to the question, “How often have you felt lonely in the last 12 months?”, using a five-point Likert scale: 1 = I never feel lonely, 2 = I rarely feel lonely, 3 = I feel lonely sometimes, 4 = I often feel lonely, and 5 = I feel lonely all the time. Third, the responses to the question, “Do you think the amount of sleep you have gotten in the last seven days has been enough to help you recover from fatigue?” were used to determine the level of sleep satisfaction, using a five-point Likert scale: 1 = more than sufficient, 2 = very sufficient, 3 = sufficient, 4 = not enough, and 5 = not at all enough. The raw data on perceived stress, loneliness, and sleep satisfaction were analyzed without any modification.

The covariates used were sex (male or female), school level (middle school or high school), region (large city, mid-size city, or small city), academic performance (high, upper-middle, medium, low-middle, or low), self-reported family income (high, upper-middle, medium, low-middle, or low), feelings of sadness or hopelessness, suicide plans, suicidal ideation, days of physical education class, and days of physical activity of 60 min or more. For feelings of sadness or hopelessness, suicide plans, and suicidal thoughts, participants were asked to report their experiences over the past year (yes or no). The number of days spent participating in physical education classes each week was recorded as follows: none, once, twice, or three or more times. The number of days spent engaged in physical activity for 60 min or more each week was recorded as 0 to 7. Raw covariate data were used without modification.

### 2.3. Data Analysis

SPSS for Windows (version 23; IBM Corp., Armonk, NY, USA) was used for the statistical analysis. First, we analyzed the characteristics of the study participants. Second, by using chi-square analysis, we examined whether the variables were statistically significant according to the number of teams that adolescents participated in regularly. Third, by using multivariate logistic regression analysis, we analyzed the association between the number of teams and the perceived stress, loneliness, and sleep satisfaction. The results are presented as odds ratios (ORs), 95% confidence intervals (CIs), and *p*-values. The statistical significance was set at *p* < 0.05.

## 3. Results

### 3.1. Frequency Analysis of Participant Characteristics

Participant characteristics are listed in Table 1. Among the 1393 participants, 525 (37.7%) and 868 (62.3%) were male and female, respectively. Academic performance was high, upper-middle, medium, low-middle, and low for 163 (11.7%), 264 (18.9%), 348 (25%), 356 (25.6%), and 262 (18.8%) participants, respectively. Family income was high, upper-middle, medium, low-middle, and low for 198 (14.2%), 346 (24.8%), 550 (39.5%), 187 (13.4%), and 112 (8.1%) participants, respectively. Feelings of sadness and hopelessness were reported by 328 (23.5%) and 1065 (76.5%) participants, respectively. No suicide plans were reported by 583 (41.9%) and 810 (58.1%) participants. Suicidal ideation was absent in 180 (12.9%) and present in 1213 (87.1%) participants who had attempted suicide. Participants were also able to identify their suicidal plans and thoughts. The number of days spent participating in physical education classes per week was three for 458 participants (32.9%), two for 277 participants (27.1%), one for 226 participants (16.2%), and zero for 332 participants (23.8%). Thus, the proportion of adolescents who participated in sports activities three or more times a week was the highest. On the other hand, the highest proportion of students reported spending zero days per week engaged in physical activity for more than 60 min (403 participants [28.9%]). Participation in zero, one, two, and three team(s) was reported by 627 (45%), 413 (29.7%), 205 (14.7%), and 77 students (5.5%), respectively.

### 3.2. Chi-Square Analysis

The results of the chi-square analysis of the differences according to the number of teams are shown in Table 2. Specifically, sex (χ2 = 23.219, *p* < 0.001), school level (χ2 = 262.955, *p* < 0.001), academic performance (χ2 = 34.498, *p* = 0.005), family income (χ2 = 32.856, *p* = 0.008), suicide plan (χ2 = 17.241, *p* = 0.002), loneliness (χ2 = 41.839, *p* < 0.001), perceived stress (χ2 = 201.686, *p* < 0.001), sleep satisfaction (χ2 = 47.623, *p* < 0.001), number of days spent participating in physical education classes per week (χ2 = 213.04, *p* < 0.001), and days of physical activity for more than 60 min per week (χ2 = 126.664, *p* < 0.001) were identified as significant.

### 3.3. The Association between the Number of Teams and Perceived Stress

Table 3 shows results of the multivariate logistic regression analysis of the association between the number of teams a student regularly participates in and perceived stress. Among those who belonged to one team, the OR for feeling very much stressed was 0.613 (range: 0.387–0.969; *p* = 0.036). Among those who belonged to two teams, the OR for feeling very much stressed was 0.482 (range: 0.281–0.286; *p* = 0.008). Among those who belonged to two teams, the OR for feeling a lot of stress was 0.514 (range: 0.304–0.870; *p* = 0.013), and that for not feeling much stress was 2.663 (range: 1.103–6.426; *p* = 0.029). The OR for not feeling much stress was 4.697 for those who participated in three teams (range: 1.531–14.408; *p* = 0.007) and 6.671 for those who participated in four or more teams (range: 1.858–23.953; *p* = 0.004). The OR for feeling no stress at all was 11.629 (range: 2.229–60.661; *p* = 0.004) for those participating in three teams and 93.531 (range: 19.260–454.207; *p* < 0.001) for those participating in four or more teams. These findings indicate that participants were less likely to perceive stress when they participated in sports activities than when they did not. In other words, regularly participating in a greater number of teams was more strongly associated with a reduction in perceived stress.

### 3.4. Association between the Number of Teams a Student Regularly Participates in and Loneliness

Table 4 shows the results of the multivariate logistic regression for the association between the number of teams that a student regularly participates in and loneliness (with ORs and 95% CIs). The OR for rarely feeling lonely was 2.651 (range: 1.148–6.123; *p* = 0.022) among those who belonged to one team. The OR for feeling lonely all the time was 0.37 (range: 0.155–0.884; *p* = 0.025) among those who belonged to two teams. This finding indicates that students were less likely to feel lonely when they were on two teams than when they were on none. In other words, participating in a greater number of teams was associated with a decrease in loneliness.

### 3.5. The Association between the Number of Teams a Student Regularly Participates in and Sleep Satisfaction

Table 5 shows the results of the multivariate logistic regression for the association between the number of teams a student regularly participates in and sleep satisfaction (with ORs and 95% CIs). The OR for very sufficient sleep was 4.371 (range: 1.627–11.742; *p* = 0.003) for those who participated in four or more teams. These findings suggest that participating in a greater number of sports teams was associated with increased sleep satisfaction.

## 4. Discussion

In this study, we examined the associations between the number of school sports teams that adolescents regularly participate in and factors such as perceived stress, loneliness, and sleep satisfaction among Korean adolescents who have attempted suicide. Our results indicated that a greater participation in team sports at school was associated with a reduction in perceived stress/loneliness and an increase in sleep satisfaction. First, there was a strong association between the number of teams and reduced perceived stress among Korean adolescents who have attempted suicide, thus suggesting that participating in school sports activities reduced perceived stress levels. This is partly consistent with previous research showing that school sports help reduce perceived stress in adolescents [4,19]. In addition to reducing perceived stress, physical activity has been shown to reduce anxiety, depression, and alcohol abuse—which contribute to suicide attempts—and may in turn help prevent suicide [4]. Murray et al. [19] reported that adolescents who participate in team sports have lower perceived stress and higher levels of perceived stress coping than those who have never participated, thus suggesting that participation in team sports plays an important role in promoting better mental health. In addition, Teh and Krishnan-Vasanthi [20] reported lower perceived stress among young athletes involved in team sports than among athletes who participated in individual sports. However, no study has reported a direct effect of participation in team sports on perceived stress among adolescents who have attempted suicide. Together, the available evidence suggests that, for adolescents with a history of suicidal behaviors, participation in team sports can reduce perceived stress and suicide-related risk behaviors.

Second, there was a strong association between the number of teams and reduced loneliness among Korean adolescents who have attempted suicide; this finding is in accordance with those of Santos et al. [21] and Pinto et al. [22], who reported that adolescent participation in physical activity and physical education classes contributes to reduced loneliness. Santos et al. [21] reported that female adolescents who participated in physical education classes were less likely to feel lonely and more likely to have a greater number of friends than those who did not participate. Pinto et al. [22] argued that physical education classes can help solve the problems of adolescents who feel lonely because adolescents who do not participate in physical education classes are more likely to feel lonely. Loneliness is a predictor of suicidal behavior, and several studies have reported that adolescent participation in physical activity is associated with reduced loneliness or suicidal behaviors [5,23]. Southerland et al. [5] reported that participation in team sports helped reduce suicide-related risk behaviors such as suicidal ideation, planning, and attempts among middle school students. In other words, school sports may act as a mediator in the effect of physical activity on loneliness and suicide-related behaviors.

Third, there was a strong association between the number of school sports teams in which adolescents regularly participate and sleep satisfaction among Korean adolescents who have attempted suicide. Several studies have reported that physical activity positively affects sleep [24,25]. Dolezal et al. [24] conducted a systematic review of the relationship between physical activity and sleep and found that, although there were differences based on sex, age, and exercise type, physical activity was beneficial for sleep quality. Baldursdottir et al. [25] partially supported this finding, reporting that the more physically active adolescents were, the higher their sleep quality. In particular, team sports had a greater positive effect on sleep quality than individual sports activities [13], and non-participation in team sports and sleep deprivation were highly correlated with increased suicide-related behaviors among adolescents [26]. In other words, adolescent participation in team sports activities is more likely to reduce suicidal behaviors and improve sleep quality [13,18,24,25,26]. Therefore, at-risk adolescents with a history of suicidal ideation, suicide plans, and suicide attempts may be able to prevent recurrence by improving sleep quality via physical education and team sports activities.

Our results indicated that participating in a greater number of sports teams at school was strongly associated with reduced perceived stress, reduced loneliness, and increased sleep satisfaction. This suggests that adolescents who have attempted suicide should be encouraged to actively participate in physical education activities because they are likely to influence suicide-related behavioral variables. In other words, school sports may represent a strategy for follow-up management and the prevention of suicide recurrence among adolescents who have attempted suicide. Despite the positive effects of team sports activities on adolescents in this study, 45% of the participants did not participate in team sports. Therefore, it is necessary to create environments that promote the participation of adolescents who have attempted suicide in schools, teams, and sports. One way to achieve this is to revitalize school sports clubs that operate within the scope of the curriculum. School sports clubs in South Korea are organized around team competitions such as soccer, basketball, and badminton, and are operated by adolescents opening or joining clubs of their choice [27]. The way sports clubs are run is conducive to adolescents who have attempted suicide; therefore, it would be helpful to use them. Physical education teachers should develop and operate sports club programs that reflect the characteristics of adolescents who have attempted suicide. Physical education teachers need to design the entire program in such a way that it provides an opportunity for adolescents who have attempted suicide to develop the strength to overcome various difficulties through sports clubs and to reduce negative thoughts. For example, while enjoying team sports with friends at a sports club, the program could be designed to allow students to experience the process of relieving depressed moods caused by career paths or various personal worries, recognizing their own existence, restoring self-esteem, and experiencing the desire for physical activity in the process of sweating and communicating with peers [28]. Through this learning, adolescents who have attempted suicide will be able to develop the strength to cope with difficult situations and develop the confidence to fend for themselves [10]. This leads adolescents to attempt “living” rather than making the negative choice to attempt “suicide”.

This study has several limitations. First, as this cross-sectional study used data from the 2022 KYRBS, there are limitations in the data’s ability to explain temporal or causal relationships. Hence, this study did not predict suicidal ideation and planning based on the demographics or intervening variables. Moreover, because this study is cross-sectional, the medical outcomes are unclear, and it is difficult to find a direct correlation. However, we only wanted to confirm the relationship between the variables and increase their validity by comparing the results with previous studies. In the future, more well-designed studies should conduct an in-depth analysis of the hypothesis. Second, perceived stress, loneliness, and sleep satisfaction were self-reported based on the subjective judgments of the adolescents who participated rather than objective values. These reports can be influenced by an individual’s physical condition and mood at the time of the survey. Therefore, even if the same person responds to the survey, the survey results may vary depending on the situation. Third, this study did not use validated scales to record the data related to stress and loneliness, which can undermine the reliability of the research. Nonetheless, this study is valuable in that it confirmed the potential of school and team sports as tools for preventing suicide recurrence. The results will also contribute to the development of follow-up strategies and policies related to suicide prevention among adolescents.

## 5. Conclusions

Our results indicated that a greater participation in team sports at school was associated with a reduction in perceived stress/loneliness and an increase in sleep satisfaction among Korean adolescents who have attempted suicide. This suggests that participating in school sports, and team sports in particular, can help prevent suicide recurrence among adolescents. In addition to their effects on perceived stress, loneliness, and sleep satisfaction, school sports are an advantageous tool for suicide prevention given their low costs, absence of side effects, and ease of participation. Therefore, school sports should be emphasized in follow-up and management strategies for preventing the recurrence of suicidal behavior among adolescents.

## Figures and Tables

**Table 1:** Participant characteristics (*n* = 1393).

Variables		Total (%)
Sex	Male	525 (37.7%)
	Female	868 (62.3%)
School level	Middle school	839 (60.2%)
	High school	554 (39.8%)
Region	Large cities	657 (47.2%)
	Mid-size cities	661 (47.4%)
	Small cities	75 (5.4%)
Academic performance	High	163 (11.7%)
	Upper-middle	264 (18.9%)
	Medium	348 (25%)
	Low-middle	356 (25.6%)
	Low	262 (18.8%)
Family income	High	198 (14.2%)
	Upper-middle	346 (24.8%)
	Medium	550 (39.5%)
	Low-middle	187 (13.4%)
	Low	112 (8.1%)
Feelings of sadness or hopelessness	No	328 (23.5%)
	Yes	1065 (76.5%)
Suicide plans	No	583 (41.9%)
	Yes	810 (58.1%)
Suicidal ideation	No	180 (12.9%)
	Yes	1213 (87.1%)
Days of physical education class	Three or more times a week	458 (32.9%)
	Twice a week	377 (27.1%)
	Once a week	226 (16.2%)
	None	332 (23.8%)
Days of physical activity of 60 min or more	None	403 (28.9%)
	1 day a week	195 (14%)
	2 days a week	211 (15.2%)
	3 days a week	203 (14.6%)
	4 days a week	112 (8%)
	5 days a week	95 (6.8%)
	6 days a week	32 (2.3%)
	7 days a week	142 (10.2%)
Number of school sports teams in which the student regularly participates	Four or more teams	77 (5.5%)
	Three teams	71 (5.1%)
	Two teams	205 (14.7%)
	One team	413 (29.7%)
	None	627 (45%)

**Table 2:** Differences between variables depending on the number of school sports teams in which the student regularly participates.

Variables		Four or More Teams	Three Teams	Two Teams	One Team	None	χ2 (*p*)
Gender	Male	43 (8.2%)	36 (6.8%)	88 (16.8%)	139 (26.5%)	219 (41.7%)	23.219 (<0.001 ***)
	Female	34 (3.9%)	35 (4%)	117 (13.5%)	274 (31.6%)	408 (47%)	
School level	Middle school	52 (6.2%)	53 (6.3%)	165 (19.7%)	337 (40.2%)	232 (27.6%)	262.955 (<0.001 ***)
	High school	25 (4.5%)	18 (3.3%)	40 (7.2%)	76 (13.7%)	395 (71.3%)	
Region	Large cities	38 (5.8%)	33 (5%)	101 (15.4%)	182 (27.7%)	303 (46.1%)	5.696 (0.681)
	Middle-size cities	33 (5%)	32 (4.8%)	92 (13.9%)	210 (31.8%)	294 (44.5%)	
	Small cities	6 (8%)	6 (8%)	12 (16%)	21 (28%)	30 (40%)	
Academic performance	High	18 (11%)	16 (9.8%)	19 (11.7%)	46 (28.2%)	64 (39.3%)	34.498 (0.005 **)
	Upper-middle	13 (4.9%)	14 (5.3%)	49 (18.6%)	75 (28.4%)	113 (42.8%)	
	Medium	14 (4%)	15 (4.3%)	60 (17.2%)	114 (32.8%)	145 (41.7%)	
	Low-middle	16 (4.5%)	15 (4.2%)	48 (13.5%)	104 (29.2%)	173 (48.6%)	
	Low	16 (6.1%)	11 (4.2%)	29 (11.1%)	74 (28.2%)	132 (50.4%)	
Family income	High	19 (9.6%)	17 (8.6%)	31 (15.6%)	54 (27.3%)	77 (38.9%)	32.856 (0.008 **)
	Upper-middle	16 (4.6%)	20 (5.8%)	60 (17.3%)	100 (28.9%)	150 (43.4%)	
	Medium	22 (4%)	20 (3.6%)	75 (13.7%)	181 (32.9%)	252 (45.8%)	
	Low-middle	8 (4.3%)	10 (5.4%)	24 (12.8%)	52 (27.8%)	93 (49.7%)	
	Low	12 (10.7%)	4 (3.6%)	15 (13.4%)	26 (23.2%)	55 (49.1%)	
Feelings of sadness or hopelessness	No	17 (5.2%)	24 (7.3%)	50 (15.3%)	108 (32.9%)	129 (39.3%)	8.954 (0.062)
	Yes	60 (5.6%)	47 (4.4%)	155 (14.6%)	305 (28.6%)	498 (46,8%)	
Suicide plans	No	25 (4.3%)	35 (6%)	87 (14.9%)	201 (34.5%)	235 (40.3%)	17.241 (0.002 **)
	Yes	52 (6.4%)	36 (4.4%)	118 (14.6%)	212 (26.2%)	392 (48.4%)	
Suicidal ideation	No	10 (5.6%)	11 (6.1%)	35 (19.4%)	59 (32.8%)	65 (36.1%)	7.878 (0.096)
	Yes	67 (5.5%)	60 (5%)	170 (14%)	354 (29.2%)	562 (46.3%)	
Loneliness	Never feel lonely	5 (6.8%)	5 (6.8%)	14 (18.9%)	14 (18.9%)	36 (48.6%)	41.839 (0.001 ***)
	Rarely feel lonely	9 (7%)	8 (6.3%)	22 (17.2%)	51 (39.8%)	38 (29.7%)	
	Feel lonely sometimes	17 (4.3%)	18 (4.5%)	65 (16.2%)	136 (34%)	164 (41%)	
	Feel lonely often	23 (4.9%)	20 (4.3%)	76 (16.3%)	128 (27.5%)	219 (47%)	
	Feel lonely all the time	23 (7.1%)	20 (6.2%)	28 (8.6%)	84 (25.8%)	170 (52.3%)	
Perceived stress	Feel very much	29 (4.7%)	25 (4%)	76 (12.3%)	163 (26.4%)	325 (52.6%)	201.686 (0.001 ***)
	Feel a lot	16 (3.4%)	20 (4.3%)	69 (14.9%)	160 (34.6%)	198 (42.8%)	
	Feel a little	7 (3.4%)	12 (5.8%)	41 (19.8%)	65 (31.4%)	82 (39.6%)	
	Do not feel much	7 (10%)	9 (12.8%)	16 (22.9%)	20 (28.6%)	18 (25.7%)	
	Do not feel at all	18 (51.4%)	5 (14.3%)	3 (8.6%)	5 (14.3%)	4 (11.4%)	
Sleep satisfaction	Very sufficient	11 (14.9%)	8 (10.8%)	6 (8.1%)	13 (17.6%)	36 (48.6%)	47.623 (0.001 ***)
	Sufficient	6 (5.5%)	5 (4.6%)	19 (17.4%)	36 (33%)	43 (39.5%)	
	Just so	17 (5.3%)	17 (5.3%)	60 (18.7%)	99 (30.8%)	128 (39.9%)	
	Not enough	16 (4%)	22 (5.5%)	64 (16%)	134 (33.5%)	164 (41%)	
	Not at all enough	27 (5.5%)	19 (3.9%)	56 (11.4%)	131 (26.8%)	256 (52.4%)	
Days of physical education class	Three or more times a week	51 (11.1%)	36 (7.9%)	101 (22%)	167 (36.5%)	103 (22.5%)	213.04 (0.001 ***)
	Twice a week	14 (3.7%)	19 (5%)	61 (16.2%)	114 (30.3%)	169 (44.8%)	
	Once a week	8 (3.5%)	9 (4%)	20 (8.9%)	68 (30.1%)	121 (53.5%)	
	None	4 (1.2%)	7 (2.1%)	23 (6.9%)	64 (19.3%)	234 (70.5%)	
Days of physical activity of 60 min or more	None	19 (4.7%)	7 (1.7%)	47 (11.7%)	87 (21.6%)	243 (60.3%)	126.664 (0.001 ***)
	1 day a week	7 (3.6%)	7 (3.6%)	23 (11.8%)	59 (30.2%)	99 (50.8%)	
	2 days a week	13 (6.2%)	10 (4.7%)	34 (16.1%)	62 (29.4%)	92 (43.6%)	
	3 days a week	7 (3.4%)	14 (6.9%)	32 (15.8%)	61 (30.1%)	89 (43.8%)	
	4 days a week	8 (7.1%)	6 (5.4%)	24 (21.4%)	44 (39.3%)	30 (26.8%)	
	5 days a week	4 (4.2%)	7 (7.4%)	15 (15.8%)	45 (47.4%)	24 (25.2%)	
	6 days a week	3 (9.3%)	6 (18.8%)	4 (12.5%)	13 (40.6%)	6 (18.8%)	
	7 days a week	16 (11.3%)	14 (9.8%)	26 (18.3%)	42 (29.6%)	44 (31%)	

** *p* < 0.01, *** *p* < 0.001, assessed through chi-square analyses.

**Table 3:** Association between the number of teams a student regularly participates in and perceived stress.

Variables		Perceived Stress Odds Ratios (95% Confidence Intervals)			
		Feel Very Much	Feel a Lot	Do Not Feel Much	Do Not Feel at All
Number of teams a student regularly participates in	Four or more teams	0.995 (0.384–2.582) *p* = 0.992	0.704 (0.265–1.868) *p* = 0.481	6.671 (1.858–23.953) *p* = 0.004 **	93.531 (19.260–454.207) *p* < 0.001 ***
	Three teams	0.602 (0.260–1.394) *p* = 0.236	0.569 (0.251–1.291) *p* = 0.178	4.697 (1.531–14.408) *p* = 0.007 **	11.629 (2.229–60.661) *p* = 0.004 **
	Two teams	0.482 (0.281–0.826) *p* = 0.008 **	0.514 (0.304–0.870) *p* = 0.013 *	2.663 (1.103–6.426) *p* = 0.029 *	2.025 (0.362–11.326) *p* = 0.422
	One team	0.613 (0.387–0.969) *p* = 0.036 *	0.758 (0.484–1.187) *p* = 0.225	2.245 (0.996–5.061) *p* = 0.051	2.119 (0.499–9.001) *p* = 0.309
	None (reference)	1	1	1	1

* *p* < 0.05, ** *p* < 0.01, *** *p* < 0.001, multivariate logistic regression analysis.

**Table 4:** Association between the number of teams a student regularly participates in and loneliness.

Variables		Loneliness Odds Ratios (95% Confidence Intervals)			
		Rarely Feel Lonely	Feel Lonely Sometimes	Feel Lonely Often	Feel Lonely All the Time
Number of teams a student regularly participates in	4 or more teams	0.943 (0.252–3.523) *p* = 0.93	0.508 (0.156–1.648) *p* = 0.259	0.783 (0.241–2.540) *p* = 0.684	1.199 (0.359–4.009) *p* = 0.768
	3 teams	0.872 (0.231–3.286) *p* = 0.839	0.556 (0.173–1.788) *p* = 0.324	0.721 (0.220–2.359) *p* = 0.589	1.056 (0.310–3.603) *p* = 0.930
	2 teams	1.068 (0.430–2.650) *p* = 0.888	0.718 (0.328–1.575) *p* = 0.409	0.777 (0.350–1.722) *p* = 0.534	0.37 (0.155–0.884) *p* = 0.025 *
	1 team	2.651 (1.148–6.123) *p* = 0.022 *	1.468 (0.697–3.093) *p* = 0.312	1.218 (0.572–2.593) *p* = 0.608	1.080 (0.495–2.356) *p* = 0.846
	None (reference)	1	1	1	1

* *p* < 0.05, multivariate logistic regression analysis.

**Table 5:** Association between the number of teams a student regularly participates in and sleep satisfaction.

Variables		Sleep Satisfaction Odds Ratios (95% Confidence Intervals)			
		Very Sufficient	Sufficient	Not Enough	Not at All Enough
Number of teams a student regularly participates in	Four or more teams	4.371 (1.627–11.742) *p* = 0.003 **	0.927 (0.314–2.741) *p* = 0.891	0.891 (0.409–1.942) *p* = 0.772	1.527 (0.735–3.171) *p* = 0.256
	Three teams	2.544 (0.909–7.123) *p* = 0.075	0.868 (0.286–2.632) *p* = 0.802	1.176 (0.573–2.415) *p* = 0.658	0.944 (0.442–2.019) *p* = 0.883
	Two teams	0.471 (0.174–1.274) *p* = 0.138	0.889 (0.452–1.750) *p* = 0.734	0.901 (0.566–1.435) *p* = 0.660	0.734 (0.454–1.186) *p* = 0.206
	One team	0.578 (0.270–1.239) *p* = 0.159	1.089 (0.612–1.936) *p* = 0.773	1.055 (0.712–1.563) *p* = 0.79	0.953 (0.644–1.41) *p* = 0.81
	None (reference)	1	1	1	1

** *p* < 0.01, multivariate logistic regression analysis.

## Data Availability

The data presented in this study are available in: https://www.kdca.go.kr/yhs/ (accessed on 30 November 2023).
